# Fungicide Effects on Fungal Community Composition in the Wheat Phyllosphere

**DOI:** 10.1371/journal.pone.0111786

**Published:** 2014-11-04

**Authors:** Ida Karlsson, Hanna Friberg, Christian Steinberg, Paula Persson

**Affiliations:** 1 Dept. of Crop Production Ecology, Swedish University of Agricultural Sciences (SLU), Uppsala, Sweden; 2 Dept. of Forest Mycology and Plant Pathology, SLU, Uppsala, Sweden; 3 INRA, UMR 1347 Agroécologie, Pole IPM, Dijon, France; University of Aveiro, Portugal

## Abstract

The fungicides used to control diseases in cereal production can have adverse effects on non-target fungi, with possible consequences for plant health and productivity. This study examined fungicide effects on fungal communities on winter wheat leaves in two areas of Sweden. High-throughput 454 sequencing of the fungal ITS2 region yielded 235 operational taxonomic units (OTUs) at the species level from the 18 fields studied. It was found that commonly used fungicides had moderate but significant effect on fungal community composition in the wheat phyllosphere. The relative abundance of several saprotrophs was altered by fungicide use, while the effect on common wheat pathogens was mixed. The fungal community on wheat leaves consisted mainly of basidiomycete yeasts, saprotrophic ascomycetes and plant pathogens. A core set of six fungal OTUs representing saprotrophic species was identified. These were present across all fields, although overall the difference in OTU richness was large between the two areas studied.

## Introduction

The phyllosphere, defined as the total above-ground parts of plants, provides a habitat for many microorganisms [1]. Phyllosphere microorganisms, including fungi, have been shown to perform important ecological functions and can be both beneficial and harmful to their host plant [2]. In agricultural crops, some phyllosphere fungi are important pathogens, while others have antagonistic properties [3] or can influence the physiology of the plant [4]. Understanding the influence of agricultural practices on phyllosphere fungal communities is important in order to create the best conditions for crop development.

Wheat is one of the most important crops worldwide and the wheat-associated fungal community was one of the first phyllosphere communities to be studied [5]. The wheat phyllosphere has been found to contain many basidiomycete yeasts such as *Cryptococcus* spp., *Sporobolomyces roseus* and filamentous saprotrophs, e.g. *Cladosporium* spp., *Alternaria* spp., *Epicoccum* spp., and plant pathogens [5]–[8]. Fungi can be present both as epiphytes and endophytes on wheat leaves. This is reflected in the different sets of fungi retrieved when washed leaf pieces are cultured compared with leaf wash liquid [9]. The main components of the fungal wheat leaf community differ in studies conducted at different sites and at different times and the mechanisms that lie behind the dynamics of fungal communities in the phyllosphere of agricultural crops are not well understood.

Plant pathogens are an important and well-studied group of wheat-associated microorganisms. Important fungal wheat leaf diseases world-wide include different types of rusts (*Puccinia* spp.), powdery mildew (*Blumeria graminis*) and leaf blotch diseases such as septoria tritici blotch (*Mycosphaerella graminicola* (*Zymoseptoria tritici*)). Septoria tritici blotch has been one of the most serious diseases of European wheat since the early 1980 s, causing up to 50% yield losses [10].

Foliar fungicides are routinely used in conventional agriculture to control fungal diseases. However, besides the desired effect on fungal pathogens, non-target fungi are also subjected to the fungicide treatment. It is important to understand the effect of fungicides on non-target fungi given the antagonistic potential of some phyllosphere fungi and interactions between different pathogens [1], [11]. Applying a fungicide to control one pathogen might even increase the problems with another, as has been shown for *Fusarium* spp. causing fusarium head blight in cereals [12], [13]. It has been hypothesised that fungicides suppress saprotrophic fungi that otherwise would act as competitors against *Fusarium* [13]. On the other hand, phyllosphere saprotrophs have been shown to accelerate leaf senescence, which could explain some of the yield increase after fungicide treatment not explained by attack of pathogens [9], [14]. More knowledge on the effect of fungicides on phyllosphere fungal communities is important in order to optimise fungicide application strategies.

Fungicides have different modes of action and can be both broad range or target a specific group of fungi [15] and the fungicide type and use vary for different crops. Previous studies examining fungicide effects on non-target fungi in the wheat phyllosphere using culture-dependent methods have shown that fungicides with different modes of action have differing effects on individual fungal taxa [7]–[9], [16]–[18]. Some of the biases of culture-dependent methods can be overcome using DNA-based methods. Recently, high-throughput sequencing technologies have revolutionized the study of microbial diversity in the phyllosphere. Consequently, knowledge on bacterial phyllosphere communities on agricultural crops is growing, but less is known about fungi [19]. So far, fungicide effects on fungal communities in the phyllosphere has only been investigated to a limited extent using DNA-based fingerprinting methods [20], [21] and high-throughput sequencing [22], but none of these studies focused on cereals.

The aims of this study were: 1) to identify the fungal community in the wheat phyllosphere using 454 high-throughput sequencing, 2) to study the effect of fungicides on fungal community composition in the wheat phyllosphere, and 3) to study differences between phyllosphere fungal communities in two areas characterised by different climate conditions and agricultural management regimes. Fungicide-treated and non-fungicide treated leaves were sampled from winter wheat fields in two areas in Sweden and fungal community composition on the leaves was analysed by amplification and 454-sequencing of the fungal ITS2 region of the ribosomal DNA.

## Materials and Methods

### Ethics statement

Permission from the farmers was obtained through the Plant Protection Centres of the Swedish Board of Agriculture in Skara (for the Northern area) and Alnarp (for the Southern area) respectively. The study did not involve any protected or endangered species.

### Sampling and plant material

**Figure 1:**
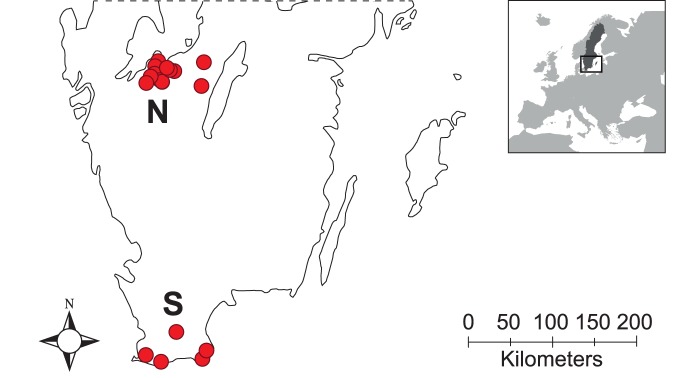
Wheat leaves were sampled in two important agricultural production areas of Sweden. Dots represent position of individual fields within in the two sampling areas. The Northern sampling area (N) is located in the Västergötland region and the Southern area (S) in the Skåne region.

Sampling of wheat fields was carried out in two important agricultural production areas of Sweden, a Northern sampling area located in the region of Västergötland and a Southern sampling area in the Skåne region (Fig. 1). The Southern area is characterised by a milder and drier climate. The two areas also differ in agricultural management, for example in terms of cropping sequence [23], the choice of wheat variety and fungicides are used more frequently in the Southern area [24]. The average winter wheat yield is about 2000 kg/ha higher in the Southern area [23]. At the time of sampling, fields in the Northern area had reached anthesis, while in the Southern area the developmental stage ranged from anthesis to the early dough ripening stage (Table 1).

**Table 1:** Distribution of wheat leaf samples between two geographical areas, fields and treatments, including weather data.

	Northern area	Southern area
Sampling date	20-June	27-June
No. of fields	13	5
No. of samples	26	16
No. of control samples	13	5
No. of fungicide-treated samples	13	11
Wheat developmental stage (DC)	61–69	61–83 nd
	06-June−20-June	13-June−27-June
Mean temp. (°C)	14.7	14.3
Mean rel. humidity (%)	81.6	82.6
Acc. rainfall (mm)	123.4	37.6
	13-June−20-June	20-June−27-June
Mean temp. (°C)	13.4	14.5
Mean rel. humidity (%)	80.5	81.4
Acc. rainfall (mm)	47.8	17.2
	19-June	26-June
Mean temp. (°C)	12.4	14.5
Mean rel. humidity (%)	93.5	78.2
Acc. rainfall (mm)	23.4	0.0

Mean temperature, mean relative humidity and accumulated rainfall^1^ are given for two weeks, one week and the day before sampling.

DC = developmental stage according to the Zadoks scale, nd = not determined for all fields.

1 Weather data from the Lantmet weather stations in Skara and Anderslöv respectively.

Wheat (*Triticum aestivum*) leaves were sampled in pest surveillance plots, disease control and variety trials placed in conventionally managed farmers’ fields during summer 2011. The pest surveillance plots are used for monitoring the incidence of pests and diseases, so fungicides or insecticides are not applied within these plots. Leaf samples representing seven different winter wheat varieties were collected from a total of 18 fields (Table 1).

All fields had received 1–3 fungicide treatments containing one or several of the following active ingredients: azoxystrobin, bixafen, cyprodinil, difenoconazole, fenpropimorph, metrafenone, picoxystrobin, prochloraz, propiconazole, prothioconazole and pyraclostrobin (see Tables S1 and S2 for further details). In fields with pest surveillance plots, fungicide application outside the plots was managed by the farmer. In field trials, fungicide application was carried out by field trial management staff.

The leaf below the flag leaf was sampled from 10 randomly chosen plants in each plot. For pest surveillance plots, plants were sampled from the fungicide-treated crop outside the plot and in the non-fungicide treated surveillance plot itself. In field trials, plants were sampled from fungicide-treated plots and from untreated control plots. However, there was no untreated plot available from one field in the Southern area. Leaves from each plot were pooled into one sample. In total, 42 samples (Table 1) and 420 leaves were used for the study. Gloves were used when picking leaves to avoid cross-contamination between fungicide-treated and untreated leaves, and between fields. The leaves were collected in clean plastic bags and stored overnight in the refrigerator and then transferred to −20°C until DNA extraction.

### DNA extraction, PCR amplification and sequencing

In order to capture both endophytic and epiphytic fungi, the whole leaf tissue was used for DNA extraction. Leaves were split into halves, with the middle vein left on the half used for extraction. The 10 halved leaves were cut into smaller pieces and placed in a plastic bag. The samples were frozen in liquid nitrogen, homogenised with a pestle and 100 mg of each sample were transferred to another bag (Bioreba AG, Switzerland). The DNA was then extracted using the DNeasy Plant Mini kit (QIAGEN AB, Sweden) according to the manufacturer’s instructions, except for the lysis buffer, for which a larger volume was used (530 µl). The DNeasy kit was used with the QiaCube (QIAGEN AB, Sweden) with the standard plant cells and tissues protocol.

The ITS2 region was amplified on a 2720 Thermal Cycler (Life Technologies, CA, USA) using the forward primer fITS7 (GTGARTCATCGAATCTTTG; [25] and the reverse primer ITS4 (TCCTCCGCTTATTGATATGC; [26]. The length of the ITS2 is variable among fungi, ranging between ∼122 and 245 bp [25]. The ITS4 primer was tagged with an 8 bp barcode. PCR was run in 50-µl reactions with 0.8 ng/µl template, 200 µM of each nucleotide, 2.75 mM MgCl_2_, forward primer at 500 nM, tagged primer at 300 nM and 0.02 U/µl polymerase (DreamTaq Green, Thermo Scientific, MA, USA) in PCR buffer. PCR conditions were 5 min at 94°C, 30–32 cycles of [30 s at 94°C, 30 s at 57°C, 30 s at 72°C] and 7 min at 72°C. The number of cycles was adapted for each sample to give weak to moderately strong bands on the agarose gel with approximately the same strength for all samples to avoid oversaturation and distortion of the PCR pool. To determine the number of cycles necessary for each sample, test runs were conducted with non-barcoded primers starting at 25 PCR cycles before samples were run with the barcoded primers.

PCR products were cleaned using AMPure (Beckman Coulter, CA, USA) according to the manufacturer’s instructions. DNA concentration was measured on a NanoDrop 1000 spectrophotometer (Thermo Scientific, MA, USA) and the samples were pooled in equimolar amounts. The sample pool was freeze-dried and sent to LGC Genomics (Germany) for adaptor ligation and sequencing on 1/16^th^ of a plate on a GS FLX Titanium sequencer (Roche, Switzerland). Demultiplexed raw sequence data were deposited in the Sequence Read Archive (http://www.ncbi.nlm.nih.gov/sra) under the accession number SRP042192.

### Bioinformatics and taxonomic assignment

The raw sequence data were analysed using the SCATA pipeline (http://scata.mykopat.slu.se; [27]). Sequences were screened for tags and primer sequences, allowing for one mismatch for the primers in addition to degenerate bases. Sequences shorter than 200 bp and those with a mean quality score lower than 20 and containing bases with a score lower than 10 were discarded. The option “extract high quality region” was used.

Sequences were clustered into operational taxonomic units (OTUs) at a clustering level that was chosen to approximate species level. The sequences passing the quality control were clustered at 1.5% dissimilarity cut-off in SCATA, using single linkage clustering with default settings (85% alignment, collapse homopolymers to 3 bp, usearch as cluster engine, miss match penalty 1, gap open penalty 0, gap extension 1 and end gap weight 0). The level of intraspecific variation in the *ITS* sequence is variable within fungi [28], and thus using a single cut-off level will not perfectly reflect biological species. However, we found 1.5% dissimilarity to be the most appropriate level in this dataset, as higher cut-off levels would group some basidiomycete species into the same OTU.

Singletons in the full dataset were removed, as many of them were considered to represent sequencing errors [29]. In addition, singletons in each sample were removed in an effort to limit the effects of tag switching [30].

We focused on taxonomically assigning the OTUs represented by at least 10 sequences globally in the dataset (67 OTUs). Some of these could be taxonomically assigned in SCATA by including reference sequences from isolates from the Fungal Biodiversity Center CBS (http://www.cbs.knaw.nl/) and from the UNITE database including ‘species hypotheses’ accessions (version 6 09.02.2014; [31]) in the clustering. Kõljalg *et al*. [31] introduced the term ‘species hypothesis’ (SH) in order to facilitate the communication of fungal taxa discovered when clustering DNA sequences at different similarity cut-offs. Stable accession codes for all species hypotheses in the UNITE database have now been introduced [31], thus facilitating comparisons among sequence-based studies of fungi.

OTUs that did not cluster with any reference sequence in SCATA were blasted against GenBank (http://www.ncbi.nlm.nih.gov/genbank) in order to find suitable reference sequences. The most abundant sequence in each OTU was used for this purpose. Taxonomic assignment was then performed with the help of neighbour-joining trees (Figs. S1 and S2). First, the OTUs were divided into basidiomycetes and ascomycetes. Then, a multiple alignment for each phylum was generated in the stand-alone version of MAFFT (v7.058b; [32]) using the G-IN-Si option. The alignments were cut so that all sequences had the same length. Subsequently, neighbour-joining trees were constructed with the ProtDist/FastDist+BioNJ option on the phylogeny.fr web service (http://www.phylogeny.fr; [33]) using 1000 bootstraps. The OTUs were assigned to the finest possible taxonomic level.

### Statistical analyses

First, the relationship between sequencing depth and OTU richness was analysed using rarefaction curves for each area and treatment, generated with Analytical Rarefaction 1.3 (Steven M. Holland 2003, http://strata.uga.edu/software/anRareReadme.html) in steps of 1000 specimens. The relationship between the number of samples and OTU richness was analysed with species accumulation curves. The distribution of treated and untreated samples was uneven in the two sampling areas. Therefore, when more than two samples were taken per field, one fungicide-treated and one untreated sample were randomly chosen from each field for inclusion in the analysis. Species accumulation curves were generated using the *specaccum* function with the random method in the ‘Vegan’ package (version 2.0–10; [34]) in R (version 3.0.2)

As the number of sequences per sample was unequal, the dataset was rarefied to 197 sequences per sample, which was the size of the smallest sample. The rarefaction was performed using the *rrarefy* function in ‘Vegan’ (version 2.0–10; [34]). The rarefaction was repeated 1000 times on the sample-by-OTU table and the mean was taken over the 1000 matrices and used for subsequent analysis.

Second, we tested the effect of fungicide treatment and geographical area on OTU richness and community evenness (Pielou’s evenness index [35]) using linear mixed models (LMM). We used the *lmer* function in the ‘lme4’ R package [36]. A model including treatment, geographical area and their interaction, with field and the interaction between field and treatment as random factors was fitted to both OTU richness and evenness. Significance tests were performed with a Kenward-Roger modification for performing F-tests, the *KRmodcomp* function in the ‘pbkrtest’ package [37]. The LMM analyses were performed both on the full dataset and on a smaller dataset excluding two fields in the Southern area where the control samples were dominated by one single OTU, namely *Puccinia striiformis.*

Third, non-metric multidimensional scaling (NMDS) was used to explore the fungal community composition using the function *metaMDS* in the ‘Vegan’ package [34] in R. The NMDS was performed using Bray-Curtis dissimilarities with square root transformation and Wisconsin double standardisation. Subsequently, 95% confidence areas were fitted to the ordination using the *ordiellipse* function.

Fourth, we tested the effect of fungicide treatment and geographical area on both community composition and individual OTUs using generalised linear models (GLMs). First, a model was fitted in order to test the effect of geographical area, treatment and their interaction. The interaction was not significant and a model including field as a block factor and treatment as a fixed factor could be fitted to test the effect of the fungicide treatment in both areas. For these analyses, a GLM was fitted to each OTU using the *manyglm* function in the ‘mvabund’ package in R (version 3.8.4; [38]) using a negative binomial probability distribution. The rarefied sample-by-OTU table was input as the response variable. Next, the models were tested using the function *anova* in ‘mvabund’, providing both a multivariate test for the whole community and univariate tests for each OTU. The score test was used and the cor.type argument set to shrink to allow for correlated response among OTUs. P-values were adjusted for multiple testing. The analysis was performed both at the level of species and order.

For the NMDS, LMM and the GLM analyses, only the 67 taxonomically assigned OTUs (those represented by at least 10 sequences globally) were included, since they represented the majority of the sequences in the dataset.

## Results and Discussion

### Sequence data quality

In all, 56% of the 454 reads from the pool of 420 wheat leaves passed the SCATA quality filtering. Singletons made up 1.7% of the sequences in the filtered dataset (471 global singletons and 324 per-sample singletons) and were removed. The removal of per-sample singletons resulted in a loss of 30 OTUs represented by 2–5 sequences each. Non-fungal sequences were also removed and these constituted 3.5% of the dataset, mostly wheat sequences. Per sample, 0–45% non-fungal sequences were removed. The quality-controlled dataset contained 44 245 sequences in 42 samples. The number of sequences per sample ranged between 197–2978, with a mean of 1053 sequences per sample.

### Taxonomic composition and richness of the fungal community of wheat leaves

**Figure 2:**
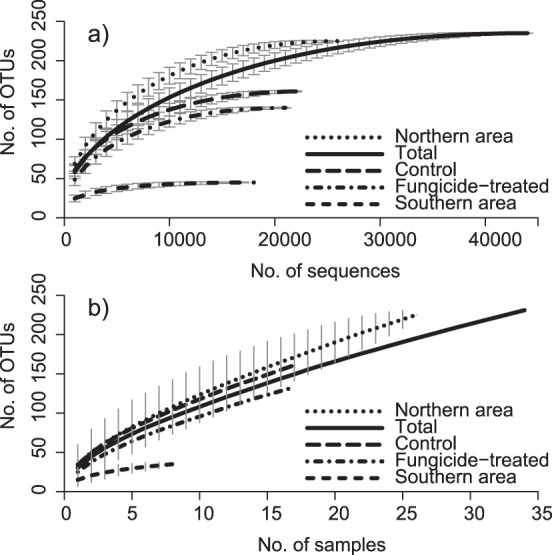
Rarefaction and species accumulation curves. a) Rarefaction curves presenting the relationship between sequencing depth and species richness in operational taxonomic units (OTUs). Error bars indicate 95% confidence intervals. b) Sample-based species accumulation curves. When more than two samples were taken per field, one fungicide-treated and one untreated sample was randomly chosen from each field for inclusion in the analysis. Error bars indicate 95% confidence intervals, only shown for Northern and Southern area respectively.

The fungal community composition in the wheat phyllosphere was characterised using 454 high-throughput sequencing. We found 235 fungal OTUs in the pool of 420 wheat leaves when clustering at 1.5% dissimilarity level. The rarefaction curves approached saturation (Fig. 2a) for all conditions, while the species accumulation curves did not (Fig. 2b).

We taxonomically assigned the OTUs containing more than 10 reads in the total dataset (67 OTUs, Table S3). These OTUs accounted for 90–100% of the sequences in the samples and none of the species in the tail were present in more than 10% of the samples. Overall, 45% of the OTUs were identified to species level and the highest taxonomic level of identification was at the order level.

**Figure 3:**
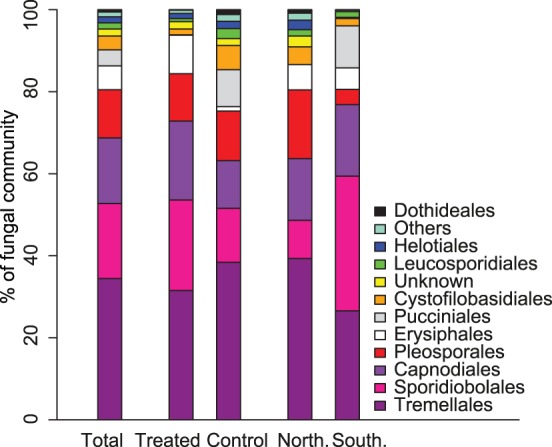
Fungal community composition on wheat leaves at the order level. Community composition is presented for the total dataset, fungicide-treated, control and samples from the Northern and Southern area respectively. Orders with low abundance have been merged to the group ‘Others’ to improve visual representation.

The fungal community in the present study consisted of almost equal proportions of ascomycetes (54%) and basidiomycetes (46%). The most common orders in the dataset were Sporidiobolales, Tremellales, Capnodiales and Pleosporales (Fig. 3).

We identified a ‘core’ fungal community of six OTUs that was found across all treatments and sites. In fact, these six most abundant OTUs were found in all samples except two, including all fields. Of these six, five were identified as basidiomycete yeasts (OTU_0_*Sporobolomyces*_*roseus*, OTU_3_*Dioszegia*_*fristingensis*, OTU_4_*Cryptococcus*_*tephrensis*, OTU_12_*Cryptococcus*_sp and OTU_9_*Dioszegia*_*hungarica*), and one as the ascomycete OTU_5_*Cladosporium*_spp. The phyllosphere fungal community of wheat has often been described as consisting of ‘pink’ yeasts (*Sporobolomyces* and *Rhodotorula* producing carotenoid pigments), ‘white’ yeasts (*Cryptococcus*) and ascomycete saprotrophs such as *Cladosporium* and *Alternaria* [17], [18]. We were able to confirm the presence of these fungal taxa previously identified by culture-dependent methods (Table 2). Comparison of DNA-based and culture-based studies may give misleading results, since culture-dependent studies typically group morphologically similar species into one category. Thus, comparing the fungal community composition at the genus level might overestimate the similarity to previous studies of the phyllosphere of wheat. Using high-throughput sequencing, we were able to describe the fungal community in more detail. Blixt et al. (2010) [6] identified16 fungal species from wheat leaves in Sweden using cloning and sequencing, 13 of these were found among the 235 OTUs in our study.

**Table 2:** Taxonomic and functional assignment of the 30 most abundant operational taxonomic units (OTUs).

OTU ID	No. of reads	Taxonomic assignment	UNITE SH-accesssion	SH %^2^	Phylum	Class	Order	Putative functional assignment [64], [65]
OTU_0	8190	*Sporobolomyces roseus*	SH196706.06FU	98.5	Basidiomycota	Microbotryomycetes	Sporidiobolales	yeast
OTU_3	5475	*Dioszegia fristingensis*	SH196962.06FU	98.5	Basidiomycota	Tremellomycetes	Tremellales	yeast
OTU_5	4760	*Cladosporium* spp	–	–	Ascomycota	Dothideomycetes	Capnodiales	saprotroph, pathogen
OTU_4	3510	*Cryptococcus tephrensis*	SH198056.06FU	98.5	Basidiomycota	Tremellomycetes	Tremellales	yeast
OTU_12	2848	*Cryptococcus* spp	SH154124.06FU	97.5	Basidiomycota	Tremellomycetes	Tremellales	yeast
OTU_9	2330	*Dioszegia hungarica*	SH196961.06FU	98.5	Basidiomycota	Tremellomycetes	Tremellales	yeast
OTU_1	2301	*Puccinia striiformis*	SH205903.06FU	98.5	Basidiomycota	Pucciniomycetes	Pucciniales	pathogen
OTU_14	2223	*Mycosphaerella graminicola*	SH044710.06FU	98	Ascomycota	Dothideomycetes	Capnodiales	pathogen
OTU_13	2012	*Blumeria graminis*	SH195226.06FU	98.5	Ascomycota	Leotiomycetes	Erysiphales	pathogen
OTU_7	1268	*Udeniomyces pannonicus*	SH217650.06FU	98.5	Basidiomycota	Tremellomycetes	Cystofilobasidiales	yeast
OTU_6	1136	*Dioszegia* spp	SH196959.06FU	98.5	Basidiomycota	Tremellomycetes	Tremellales	yeast
OTU_24	929	*Phaeosphaeriaceae* sp1a	–	–	Ascomycota	Dothideomycetes	Pleosporales	-
OTU_27	836	*Ascochyta* sp	SH233950.06FU	98.5	Ascomycota	Dothideomycetes	Pleosporales	saprotroph, pathogen [57]
OTU_16	560	*Phaeosphaeria juncophila*	SH227803.06FU	98.5	Ascomycota	Dothideomycetes	Pleosporales	saprotroph
OTU_18	555	*Ascochyta skagwayensis*	–	–	Ascomycota	Dothideomycetes	Pleosporales	saprotroph, pathogen
OTU_26	492	*Leucosporidiella fragaria*	SH212317.06FU	98.5	Basidiomycota	Microbotryomycetes	Leucosporidiales	yeast
OTU_22	459	*Alternaria malorum*	–	–	Ascomycota	Dothideomycetes	Pleosporales	pathogen, saprotroph [66]
OTU_19	424	*Phaeosphaeriaceae* sp5a	SH227813.06FU	98.5	Ascomycota	Dothideomycetes	Pleosporales	–
OTU_47	327	*Phaeosphaeria nodorum*	SH206989.06FU	98.5	Ascomycota	Dothideomycetes	Pleosporales	pathogen
OTU_34	297	Helotiales sp1a	–	–	Ascomycota	Leotiomycetes	Helotiales	–
OTU_28	249	*Aureobasidium pullulans* a	–	–	Ascomycota	Dothideomycetes	Dothideales	saprotroph, antagonist [52]
OTU_25	179	*Leucosporidium golubevii*	SH212315.06FU	98.5	Basidiomycota	Microbotryomycetes	Leucosporidiales	yeast
OTU_50	171	Cystofilobasidiales sp a	–	–	Basidiomycota	Tremellomycetes	Cystofilobasidiales	–
OTU_32	154	*Cryptococcus wieringae*	SH216503.06FU	98.5	Basidiomycota	Tremellomycetes	Filobasidiales	yeast
OTU_35	152	Pleosporales sp1	–	–	Ascomycota	Dothideomycetes	Pleosporales	–
OTU_43	145	Helotiales sp1b	–	–	Ascomycota	Leotiomycetes	Helotiales	–
OTU_44	124	*Bullera globospora*	SH197114.06FU	98.5	Basidiomycota	Tremellomycetes	Tremellales	yeast
OTU_20	123	*Monographella* spp	SH216927.06FU	98.5	Ascomycota	Sordariomycetes	Xylariales	pathogen
OTU_33	114	*Phaeosphaeriaceae* sp5b	–	–	Ascomycota	Dothideomycetes	Pleosporales	–
OTU_70	111	*Leptospora rubella*	–	–	Ascomycota	Dothideomycetes	Incertae sedis	saprotroph [67]

Species hypothesis accession codes in the UNITE^1^ database version are indicated when available. Functional assignment as ‘pathogen’ is only used for taxa known to be pathogenic on wheat.

1 Version 6 (02.09.2014) http://unite.ut.ee/, 2 Similarity cut-off for clustering in UNITE.

The number of ITS copies has been reported to vary by an order of magnitude among different fungal species [39], [40] and within the same fungal species [41]. This will to some extent bias quantitative comparisons among different taxa. Therefore, we focused on comparing the relative abundance of each OTU in fungicide-treated and untreated samples.

### Fungicide effects on fungal community composition

**Figure 4:**
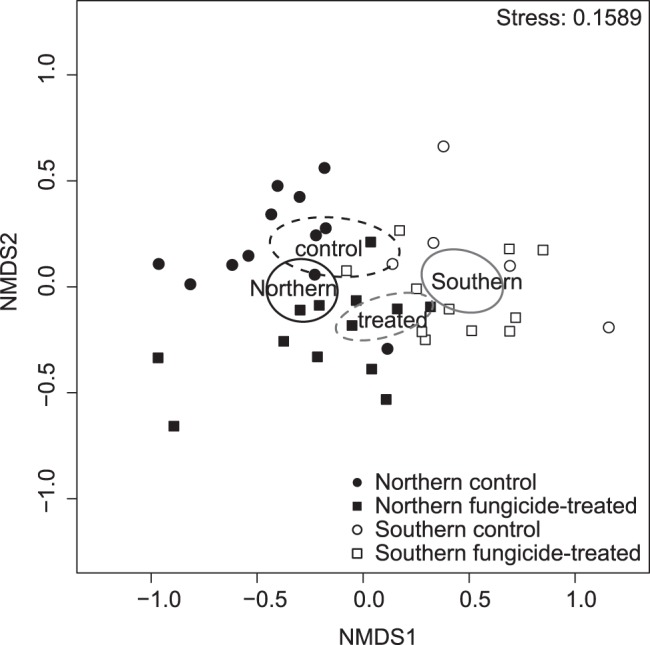
Non-metric multidimensional scaling (NMDS) of phyllosphere fungal communities of wheat. Ordination of samples with fitted environmental variables. Ellipses represent 95% confidence areas for Southern area (solid grey line), Northern area (solid black line), fungicide-treated (dashed grey line) and control (dashed black line) groups respectively. The NMDS was performed on the mean of 1000 rarefied datasets.

**Figure 5:**
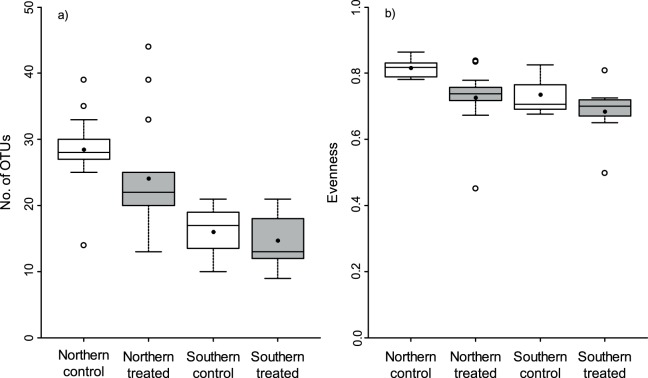
Richness of operational taxonomic units (OTUs) and community evenness. Boxplots with interquartile ranges of a) OTU richness and b) community evenness grouped by treatment (fungicide-treated and control samples) and geographical area. Horizontal lines represent medians and dots mean values. Samples from fields infected with yellow rust (*Puccinia striiformis*) in the Southern area (fields 15 and 16, Table S1) have been removed.

The application of fungicides had a significant effect on fungal community composition on wheat leaves (Fig. 4 and Table 3). The total OTU richness was lower for the fungicide-treated sample pool (Fig. 2). There was also a tendency for a lower mean OTU richness per ten leaves in the fungicide-treated samples (19.4±1.8 SE) than in the control samples (24.3±2.1 SE), but the difference was not significant (p>0.05) (Fig. 5a, Table 4). There was no interaction between fungicide treatment and geographical area for neither community composition (Table 3) nor OTU richness (p>0.05) (Table 4). When samples from fields infected with *Puccinia striiformis* were included in the analysis, the same pattern was observed (Fig. S3a, Table S4).

**Table 3:** Effect of environmental and management factors on fungal community composition in the wheat phyllosphere.

Multivariate test (anova.manyglm)				
Data	Factor	Res. Df	score	Pr(>score)
Abund	Intecept	41		
Abund	Area	40	78.29	0.001 ***
Abund	Fungicide treatment	39	67.38	0.001 ***
Abund	Area x fungicide treatment	38	22.53	0.546
Abund	Intercept	41		
Abund	Field	24	690.8	0.001 ***
Abund	Fungicide treatment	23	125	0.001 ***

Significance codes: *** = 0.001, Abund = 67 most abundant OTUs.

**Table 4:** Summary of the linear mixed model analysis of OTU richness and evenness.

OTU Richness			
Random effects	Variance	Standard deviation	
Field	16.0	4.00	
Field x fungicide treatment	0.00	0.00	
Residual	36.7	6.06	
**Fixed effects**	**Estimate**	**Standard error**	**t-value**
Intercept	28.5	2.01	14.1
Fungicide treatment (treated)	−4.39	2.38	−1.84
Area (Southern)	−12.3	4.82	−2.55
Fungicide treatment x Area	2.84	5.06	0.56
**Evenness**			
**Random effects**	**Variance**	**Standard deviation**	
Field	0.000	0.000	
Field x fungicide treatment	0.002	0.040	
Residual	0.004	0.065	
**Fixed effects**	**Estimate**	**Standard error**	**t-value**
Intercept	0.816	0.021	38.6
Fungicide treatment (treated)	−0.089	0.030	−2.99
Area (Southern)	−0.073	0.052	−1.40
Fungicide treatment x Area	0.025	0.066	0.380

Samples from fields infected with yellow rust (*Puccinia striiformis*) (fields 15 and 16, Table S1) were excluded.

Fungicide treatment affected community evenness negatively (p<0.01), and there was no interaction with geographical area (p>0.05) (Fig. 5b, Table 4). However, the pattern was different when samples from fields infected with *P*. *striiformis* were included. When these samples were included, there was a significant interaction between area and fungicide treatment (p<0.05) and evenness tended to be higher in fungicide-treated samples than in control samples in the Southern area (Fig. S3b, Table S4). *P*. *striiformis* dominated the control samples when present in a field, and consequently the evenness in these samples was very low. This dominance might have been further amplified due to the pathogenic activity of *P*. *striiformis*, physically changing the leaf surface and thus possibly making it less suitable to other fungi, or by a high amount of *P*. *striiformis* biomass masking the less abundant community members since samples were pooled in equimolar amounts [42].

**Figure 6:**
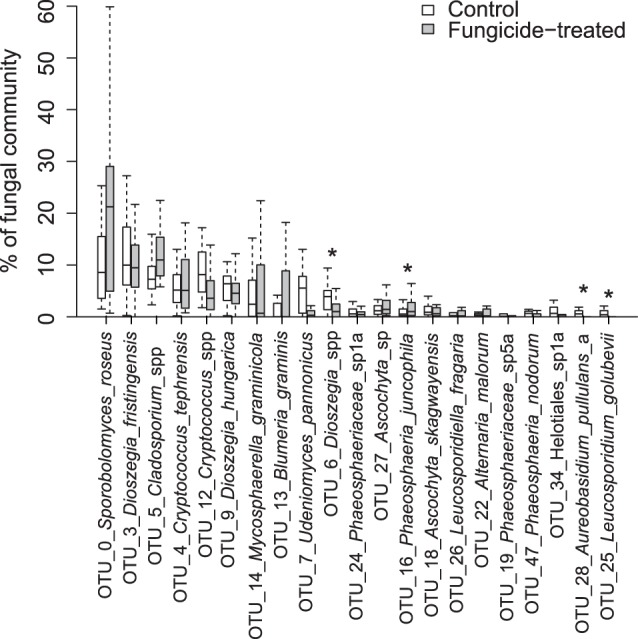
Distribution of community abundance for the most abundant OTUs grouped by treatment. Boxplots with interquartile ranges showing the relative abundances of the 21 most abundant operational taxonomic units (OTUs) in the dataset grouped by treatment. Outliers are not shown, OTU_1_*Puccinia*_*striiformis* is therefore excluded. Significant (p<0.05) differences are marked with an asterisk.

The community composition at the order level was significantly different for fungicide-treated and untreated samples (Fig. 3). The proportion of Leucosporidiales (p<0.05) and Dothideales (p<0.05) was lower in fungicide-treated samples than in the control samples. This was reflected at the species level, where univariate tests showed that the relative abundance of three OTUs: OTU_6_*Dioszegia*_sp (p<0.05), OTU_28_*Aureobasidium*_*pullulans*_a (p<0.05) and OTU_25_*Leucosporidium*_*golubevii* (p<0.05), was lower in fungicide-treated leaves than control leaves. OTU_6_*Dioszegia*_sp was similar to the *ITS* sequences of both *D*. *crocea* and *D*. *aurantiaca*. These species have been isolated from both the phyllosphere [43] and the rhizosphere of different plants [44], [45]. *Leucosporidium golubevii* is a yeast discovered in freshwater [46], and has been reported from the phyllosphere of balsam poplar [47]. In addition, the relative abundance of OTU_16_*Phaeosphaeria*_*juncophila* (p<0.01) was higher in fungicide-treated leaves (Fig. 6). *Phaeosphaeria juncophila* was first isolated from the rush *Juncus articulatus*, but little is known about its ecology.

The fungicide sensitivity of phyllosphere fungi has mostly been investigated with fungicides that are no longer used or have been prohibited in Sweden, except for some sterol biosynthesis inhibitors (SBI). SBI fungicides have been shown to have no or a moderate effect on the ‘pink’ and ‘white’ phyllosphere yeasts, with a somewhat stronger effect on ‘white’ yeasts [16]. *Aureobasidium pullulans* is reported to be sensitive to propiconazole [16] and prochloraz [17], [18] and these two fungicides and other SBI fungicides have been used in almost all the fields in this study (Table S1). The ascomycete *A*. *pullulans* is one of the most common inhabitants of the phyllosphere of many crops [48] and is also present in many other habitats [49]. *Aureobasidium pullulans* is known to be antagonistic towards necrotrophic pathogens, e.g. grey mould in strawberries [50] and powdery mildew in durum wheat [51]. The mechanism of the antagonism is hypothesised to be competition for nutrients [52]. Some strains of *A*. *pullulans* also produce a type of antibiotic called aureobasidins [53]. The antagonistic potential of biological control agents is frequently strain-specific, depending on the mechanism of antagonism. From the sequence data, we cannot make inferences about the antagonistic capacity of an OTU. Hence, it is unknown whether the reduction in the relative abundance of *A*. *pullulans* in fungicide-treated leaves has an impact on the antagonistic capacity of the fungal community.

Several OTUs were identified as common wheat pathogens in the dataset: OTU_14_*Mycosphaerella*_*graminicola,* OTU_13_*Blumeria*_*graminis,* OTU_1_*Puccinia*_*striiformis,* OTU_47_*Phaeosphaeria*_*nodorum* (*Parastagonospora nodorum*), OTU_20_*Monographella*_spp and OTU_224_*Pyrenophora*_*tritici-repentis*. Surprisingly, there was no significant effect of fungicide treatment on the relative abundance of any of these OTUs. On the contrary, there was a tendency for higher variability in the relative abundance of *M*. *graminicola* and *B*. *graminis* in treated samples (Fig. 6), and the share of *B*. *graminis* (the only member of Erysiphales) was larger in treated samples (Fig. 3). On the other hand, *P*. *striiformis* tended to dominate the fungal community in untreated samples and was nearly absent from the fungicide-treated samples from the same fields, only being present in two fields in the Southern area (Fig. 3). Fungicide resistance in common pathogens is an increasing problem and could be an explanation for the high variability in the relative abundance of the pathogens observed here. Resistance to strobilurines in *M*. *graminicola* and even more so in *B*. *graminis* is widespread in the Nordic and Baltic countries. In addition, resistance to demethylation inhibitor fungicides is increasing in both pathogens [10]. 454 sequencing is a semi-quantitative method only allowing quantification of the relative abundance of different OTUs [25]. Thus, we were unable to determine whether the fungicide treatment had an effect on absolute abundance of the pathogens.

In soil, DNA from dead fungal mycelia has been shown to degrade rapidly [54], but data on the rate of DNA degradation in the phyllosphere are lacking. It is possible that DNA from fungi killed by the fungicides might have been detected by the PCR. The difference in community composition between fungicide-treated and untreated samples may therefore be underestimated. However, the large difference in the relative abundance of *P. striiformis* between fungicide-treated and untreated samples does not support this hypothesis.

Fungicide use was significantly correlated with changes in the relative abundance of certain fungal taxa (Fig. 6). Several hypotheses can be put forward to explain the cause of these negative or positive correlations. First, a difference in fungicide sensitivity [20] can cause some taxa to decrease relative to others in the community. Secondly, a specific taxon may be affected by the fungicides indirectly through changes in the abundance of other, potentially competing, members of the community. It is also possible that taxonomic groups are closely interconnected by unknown functional interactions leading to pairwise co-occurrence or decrease in response to another underlying factor, such as fungicide-induced changes in plant physiology [55].

### Spatial variation and biogeographical patterns

There were significant differences between fungal communities on wheat leaves sampled from two different areas in Sweden (Fig. 4, Table 3). The two areas were chosen as they differed in terms of climate conditions and agricultural management. The mean OTU richness per ten leaves was significantly lower (p<0.05) in the Southern area (13.8±1.1 SE) than in the Northern (26.3±1.6 SE) (Fig. 5a, Table 4) as well as the total OTU richness in the sample pool (Fig. 2), although the Southern area was only represented by five fields. There were more fungicide-treated samples from the Southern area but the difference in overall OTU richness persisted also when comparing the same number of fungicide-treated and untreated samples in the two areas (Fig. 2b). The community evenness tended to be lower in the Southern area (Fig. 5b, Table 4), but there was no significant difference (p>0.05) when samples dominated with *P*. *striiformis* had been removed (Fig. S3b, Table S4).

The variation in community composition among fields was high, as field was a significant factor in the GLM analysis (Table 3). In addition, most of the OTUs (155 out of 235) only occurred in one sample in the dataset. For OTU richness, the variable field explained one third of the random variation, while for evenness, field did not explain any of the random variation (Table 4).

At the order level, Sporidiobolales had a significantly higher relative abundance in the Southern area (p<0.001), while Pleosporales (p<0.01), Helotiales (p<0.05) and the unassigned group of OTUs (p<0.05) were relatively more abundant in the Northern area (Fig. 3). There were many OTUs in the *Phaeosphariaceae* and in the Pleosporales that did not match any known species. These may represent undescribed fungal species, but could also reflect intragenomic variation, although this phenomenon does not seem to be wide-spread in fungi [56]. At the species level, OTU_16_*Phaeosphaeria*_*juncophila* (p<0.05) and OTU_18_*Ascochyta*_*skagwayensis* (p<0.01) were relatively more abundant in the Northern than in the Southern area. Fungi in the genus *Ascochyta* can be weak pathogens on cereals or have a saprotrophic lifestyle [57].

**Figure 7:**
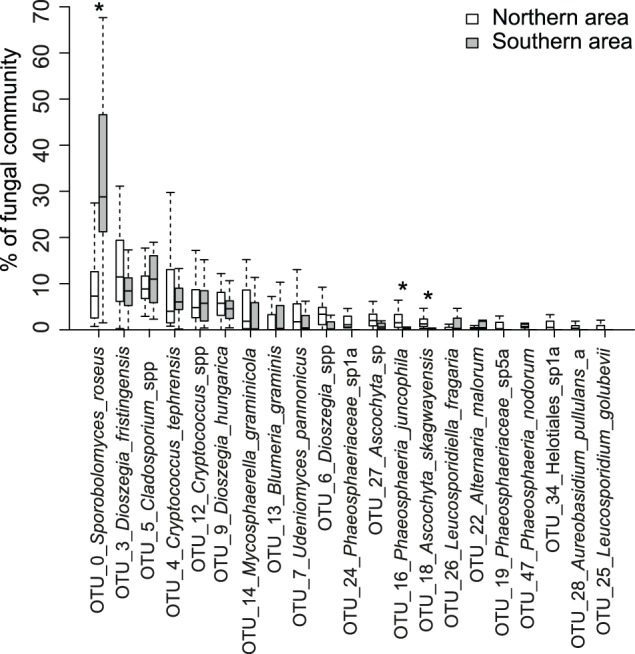
Distribution of community abundance for the most abundant OTUs grouped by geographical area. Boxplots with interquartile ranges showing the relative abundances of the 21 most abundant operational taxonomic units (OTUs) in the dataset grouped by geographical area. Outliers are not shown, OTU_1_*Puccinia*_*striiformis* is therefore excluded. Significant differences (p<0.05) are marked with an asterisk.

OTU_0_*Sporobolomyces*_*roseus* (p<0.001) was relatively more abundant in the Southern area and it was the largest community member in that area, while OTU_3_*Dioszegia*_*fristingensis* was the most abundant species in the Northern area (Fig. 7). Both of these species produce pigments and ballistospores, two characters considered to be a sign of adaptation to the phyllosphere [48]. Several studies have reported *Sporobolomyces roseus* as very common on wheat leaves [5], [7]. In contrast, Blixt *et al*. [6] only found a small proportion of this species in their study, but they selectively collected leaves diseased with *Phaeospharia nodorum*. *Dioszegia fristingensis* was described relatively recently in Germany [58], and has been reported from China [59]. It has been suggested that a group of *Dioszegia*, including *D*. *fristingensis*, is restricted to colder climates [58].

Climate is an important factor shaping phyllosphere communities. The Northern area in the present study had received more precipitation than the Southern area, and the relative humidity was higher on the day before sampling (Table 1). This could be a possible explanation for the higher fungal species richness observed in the Northern area. Levetin and Dorsey [60] found that rainfall was the most important factor for leaf surface fungi, with the number of yeasts and *Phoma* spp. correlating positively with the amount of rainfall in their study. In our study, some *Cryptococcus* and *Dioszegia* yeasts were more abundant in the Northern area, while the opposite was true for *Sporobolomyces roseus* (Fig. 7).

The atmosphere is also an important source of phyllosphere microorganisms [61]. The local air spora is one factor influencing phyllosphere community composition in different areas. Levetin and Dorsey [60] found an overlap between fungi found in the phyllosphere and the air spora. Similarly, we identified species that are commonly found in air, e.g. *Aureobasidium pullulans* and *Cladosporium* spp. [60], [62], [63]. The differences we found between areas and fields indicate that local conditions were important for fungal community composition and richness in the wheat phyllosphere in this study.

### Conclusions

Fungicide-use was associated with moderate but significant changes in fungal community composition on wheat leaves. Community evenness was negatively correlated with fungicide use. Fungicides had no effect on OTU richness on a per-plant basis, but there were fewer OTUs in the fungicide-treated sample pool. On the species level, the relative abundance of several saprotrophs was significantly affected in fungicide-treated samples. However, it is unclear whether the saprotrophic species that persist on treated leaves are capable of resisting and/or degrading the fungicides used, or what role they play in the control of pathogens and disease suppression. Interestingly, there was no significant difference in the relative abundance of common wheat pathogens, although *P*. *striiformis* tended to dominate the community in control samples when present. Further research is necessary to identify the mechanisms behind fungicide-fungi interactions in the phyllosphere of agricultural crops. Identification of the interactions between pathogenic and saprotrophic phyllosphere fungi and management practices has the potential to guide the development of sustainable disease control strategies.

## Supporting Information

Figure S1 Neighbour-joining tree of the most abundant ascomycete ITS2 sequences in the dataset. The most abundant sequence in each operational taxonomic unit (OTU_x) is included together with publicly available reference sequences and selected environmental sequences. OTUs marked with an asterisk were taxonomically assigned in SCATA. Species hypothesis accession codes in the UNITE database are given when available. Dotted lines represent branches with bootstrap values lower than 70%. *Sporobolomyces roseus* is included as an outgroup.
(EPS)

Figure S2 Neighbour-joining tree of the most abundant basidiomycete ITS2 sequences in the dataset. The most abundant sequence in each operational taxonomic unit (OTU_x) is included together with publicly available reference sequences and selected environmental sequences. OTUs marked with an asterisk were taxonomically assigned in SCATA. Species hypothesis accession codes in the UNITE database are given when available. Dotted lines represent branches with bootstrap values lower than 70%. *Rhizopus oryzae* and *Rhizopus microsporus* are included to form an outgroup.
(EPS)

Figure S3 Richness of operational taxonomic units (OTUs) and community evenness in the full dataset. Boxplots with interquartile ranges of a) OTU richness and b) community evenness grouped by treatment (fungicide-treated and control samples) and geographical area. Horizontal lines represent medians and dots mean values. Also samples from fields infected with yellow rust (*Puccinia striiformis*) in the Southern area (fields 15 and 16, Table S1) were included. F-tests with Kenward-Roger approximation showed a significant effect of geographical area on OTU richness (p<0.001) and of geographical area (p<0.01) and the interaction between treatment and area (p<0.05) on community evenness.
(EPS)

Table S1 Wheat variety, fungicide, dose and application date for wheat leaf samples collected.
(DOCX)

Table S2 Active ingredients in fungicides used in the sampled wheat fields.
(DOCX)

Table S3 Taxonomic assignment and sequence data for the 67 most abundant operational taxonomic units (OTUs) in the dataset.
(XLSX)

Table S4 Summary of the linear mixed model analysis of OTU richness and evenness including all samples.
(DOCX)
