# Supplementary material for: Fungicide Effects on Fungal Community Composition in the Wheat Phyllosphere
Source: PLoS One. 2014 Nov 4;9(11):e111786. doi: 10.1371/journal.pone.0111786 (PMC4219778; doi:10.1371/journal.pone.0111786)
Supplement: Table S1 — Wheat variety, fungicide, dose and application date for wheat leaf samples collected. (DOCX) [file pone.0111786.s004.docx]

**Table S1. Wheat variety, fungicide, dose and application date for wheat leaf samples collected.**

| **Field** | **Area** | **Wheat variety** | **Treatment** | **Date fungicide applications** | **Fungicide and dose (l/ha)** |
| --- | --- | --- | --- | --- | --- |
| 1 | Northern | Harnesk | control | - | - |
| 1 | Northern | Harnesk | treated | 5-June | Proline 0.4 + 0.17 Comet |
| 2 | Northern | Harnesk | control | - | - |
| 2 | Northern | Harnesk | treated | 15-June | Proline 0.5 + Acanto 0.25 |
| 3 | Northern | Kranich | control | - | - |
| 3 | Northern | Kranich | treated | 1-June | Tilt Top 0.48 |
| 4 | Northern | Ellvis | control | - | - |
| 4 | Northern | Ellvis | treated | 7-June | Acanto 0.25 + Armure 0.5 |
| 5 | Northern | Mulan | control | - | - |
| 5 | Northern | Mulan | treated | 6-June | Proline 0.4 + Comet 0.4 |
| 6 | Northern | Olivin | control | - | - |
| 6 | Northern | Olivin | treated | 4-June | Proline 0.4 + Sportak 0.3 + Amistar 0.2 |
| 7 | Northern | Ellvis | control | - | - |
| 7 | Northern | Ellvis | treated | 13-June | Proline 0.4 + Sportak 0.3 + Amistar 0.2 |
| 8 | Northern | Ellvis | control | - | - |
| 8 | Northern | Ellvis | treated | 31-May | Proline 0.5 + Comet 0.25 |
| 9 | Northern | Olivin | treated | - | - |
| 9 | Northern | Olivin | treated | 13-June | Acanto 0.2 + Armure 0.4 |
| 10 | Northern | Olivin | control | - | - |
| 10 | Northern | Olivin | treated | 16-June | Proline 0.5 + Comet 0.25 |
| 11 | Northern | Olivin | control | - | - |
| 11 | Northern | Olivin | treated | 15-June | Proline 0.5 + Comet 0.25 |
| 12 | Northern | Olivin | control | - | - |
| 12 | Northern | Olivin | treated | 8-June | Armur 0.6 + Acanto 0.25 |
| 13 | Northern | Ellvis | control | - | - |
| 13 | Northern | Ellvis | treated | 8-June & 12-June | Armur 0.6 + Acanto 0.25 & Proline 0.5 + Comet 0.2 |
| 14 | Southern | Gnejs | treated | 11-May | Flexity 0.25 |
| 14 | Southern | Gnejs | treated | 11-May & 31-May | Flexity 0.25 & Proline 0.4 + Comet 0.25 |
| 14 | Southern | Gnejs | treated | 11-May & 20-May & 10-June | Flexity 0.25 & Proline 0.4 + Comet 0.25 & Proline 0.4 |
| 15 | Southern | Audi | control | - | - |
| 15 | Southern | Audi | treated | 8-June | Proline 0.6 + Comet 0.25 |
| 15 | Southern | Audi | treated | 5-May & 8-June | Flexity 0.25 & Proline 0.6 + Comet 0.25 |
| 15 | Southern | Audi | treated | 5-May & 8-June | Stereo 1.0 & Proline 0.6 + Comet 0.25 |
| 16 | Southern | Audi | control | - | - |
| 16 | Southern | Audi | treated | 2-June | Proline 0.5 + Comet 0.2 + Sportak 0.3 + Tilt Top 0.3 |
| 17 | Southern | Harnesk | control | - | - |
| 17 | Southern | Harnesk | treated | 11-May & 19-May & 8-June | Flexity 0.25 + Tilt Top 0.25 & Proline 0.4 + Comet 0.25 + Sportak 0.5 & Proline 0.4 |
| 17 | Southern | Olivin | control | - | - |
| 17 | Southern | Olivin | treated | 11-May & 19-May & 8-June | Flexity 0.25 + Tilt Top 0.25 & Proline 0.4 + Comet 0.25 + Sportak 0.5 & Proline 0.4 |
| 18 | Southern | Gnejs | control | - | - |
| 18 | Southern | Gnejs | treated | 19-May & 31-May | Proline 0.4 & Proline 0.4 |
| 18 | Southern | Gnejs | treated | 19-May & 31-May | Aviator Xpro 0.63 & Aviator Xpro 0.63 |
