# Supplementary material for: Fungicide Effects on Fungal Community Composition in the Wheat Phyllosphere
Source: PLoS One. 2014 Nov 4;9(11):e111786. doi: 10.1371/journal.pone.0111786 (PMC4219778; doi:10.1371/journal.pone.0111786)
Supplement: Table S2 — Active ingredients in fungicides used in the sampled wheat fields. (DOCX) [file pone.0111786.s005.docx]

**Table S2. Active ingredients in fungicides used in the sampled wheat fields.**

| **Product** | **Producer** | **Active ingredient 1** | **g/l** | **Group^1^** | **Active ingredient 2** | **g/l** | **Group^1^** |
| --- | --- | --- | --- | --- | --- | --- | --- |
| Acanto | DuPont Sverige AB, Malmö, Sweden | picoxystrobin | 250 | QoI | - | - | - |
| Amistar | Syngenta Crop Protection A/S, Copenhagen, Denmark | azoxystrobin | 250 | QoI | - | - | - |
| Armure | Syngenta Crop Protection A/S, Copenhagen, Denmark | difenoconazole | 150 | DMI^2^ | propiconazole | 150 | DMI |
| Aviator Xpro | Bayer CropScience, Bayer AB, Staffanstorp, Sweden | prothioconazole | 150 | DMI | bixafen | 75 | SDHI |
| Comet | BASF AB Crop Protection, Trelleborg, Sweden | pyraclostrobin | 250 | QoI | - | - | - |
| Flexity | BASF AB Crop Protection, Trelleborg, Sweden | metrafenone | 300 | aryl-phenyl-ketone |  | - | - |
| Proline | Bayer CropScience, Bayer AB, Staffanstorp, Sweden | prothioconazole | 250 | DMI | - | - | - |
| Sportak | BASF AB Crop Protection, Trelleborg, Sweden | prochloraz | 450 | DMI |  | - | - |
| Stereo | Makhteshim - Agan, Leusden, Netherlands | propiconazole | 63 | DMI | cyprodinil | 250 | AP |
| Tilt Top | Makhteshim - Agan, Leusden, Netherlands | propiconazole | 125 | DMI | fenpropimorph | 375 | amines^2^ |

1 Active ingredients are grouped by their mode of action according to the FRAC Code List© 2013 (FRAC Fungicide Resistance Action Committee http://www.frac.info).

QoI = Quinone outside inhibitors, DMI = Demethylation inhibitors, SDHI = Succinate dehydrogenase inhibitors, AP = anilinopyrimidines.

2 Both these groups belong to the sterol biosynthesis inhibitors (SBI-fungicides)
