# Supplementary material for: Fungicide Effects on Fungal Community Composition in the Wheat Phyllosphere
Source: PLoS One. 2014 Nov 4;9(11):e111786. doi: 10.1371/journal.pone.0111786 (PMC4219778; doi:10.1371/journal.pone.0111786)
Supplement: Table S4 — Summary of the linear mixed model analysis of OTU richness and evenness including all samples. (DOCX) [file pone.0111786.s007.docx]

**Table S4. Summary of the linear mixed model analysis of OTU richness and evenness including all samples.**

| **OTU Richness** |  |  |  |
| --- | --- | --- | --- |
| **Random effects** | **Variance** | **Standard deviation** |  |
| Field | 15.2 | 3.89 |  |
| Field x fungicide treatment | 0.00 | 0.00 |  |
| Residual | 31.6 | 5.62 |  |
| **Fixed effects** | **Estimate** | **Standard error** | **t-value** |
| Intercept | 28.5 | 1.90 | 15.0 |
| Fungicide treatment (treated) | -4.39 | 2.20 | -1.99 |
| Area (Southern) | -14.9 | 3.67 | -4.06 |
| Fungicide treatment x Area | 4.68 | 3.87 | 1.21 |
| **Evenness** |  |  |  |
| **Random effects** | **Variance** | **Standard deviation** |  |
| Field | 0.000 | 0.000 |  |
| Field x fungicide treatment | 0.007 | 0.083 |  |
| Residual | 0.004 | 0.066 |  |
| **Fixed effects** | **Estimate** | **Standard error** | **t-value** |
| Intercept | 0.816 | 0.029 | 27.71 |
| Fungicide treatment (treated) | -0.089 | 0.042 | -2.148 |
| Area (Southern) | -0.253 | 0.059 | -4.264 |
| Fungicide treatment x Area | 0.208 | 0.079 | 2.634 |
